# New Evidence Supporting the Use of Mineralocorticoid Receptor Blockers in Drug-Resistant Hypertension

**DOI:** 10.1007/s11906-016-0643-8

**Published:** 2016-04-12

**Authors:** Hafid Narayan, David J. Webb

**Affiliations:** Royal Infirmary of Edinburgh, 51 Little France Crescent, Old Dalkeith Road, Edinburgh, EH16 4SA UK; The Queen’s Medical Research Institute, University of Edinburgh/British Heart Foundation Centre for Cardiovascular Science, 47 Little France Crescent, Edinburgh, EH16 4TJ UK

**Keywords:** Resistant hypertension, Mineralocorticoid receptor blockers, Spironolactone, Eplerenone

## Abstract

Treatment resistant hypertension (TRH), defined as a blood pressure above goal despite treatment with optimally tolerated doses of 3 antihypertensive agents of different classes, ideally including a diuretic, remains a significant problem and its management an area of uncertainty for physicians. One hypothesis is that resistant hypertension is due to abnormal sodium retention, mediated by aldosterone breakthrough occurring despite blockade of the renin-angiotensin-aldosterone system with angiotensin converting enzyme inhibitors (ACEi) or angiotensin receptor blockers (ARB). Thus, there has been renewed interest in the use of mineralocorticoid receptor blockers (MRB) to treat this condition. This article critically evaluates new evidence supporting the use of MRB in TRH published in the last 3 years. We conclude that there is now sufficient evidence to recommend MRB, in particular spironolactone, as the first choice medication to treat this condition, and for its inclusion in future guidelines.

## Introduction

**Fig. 1:**
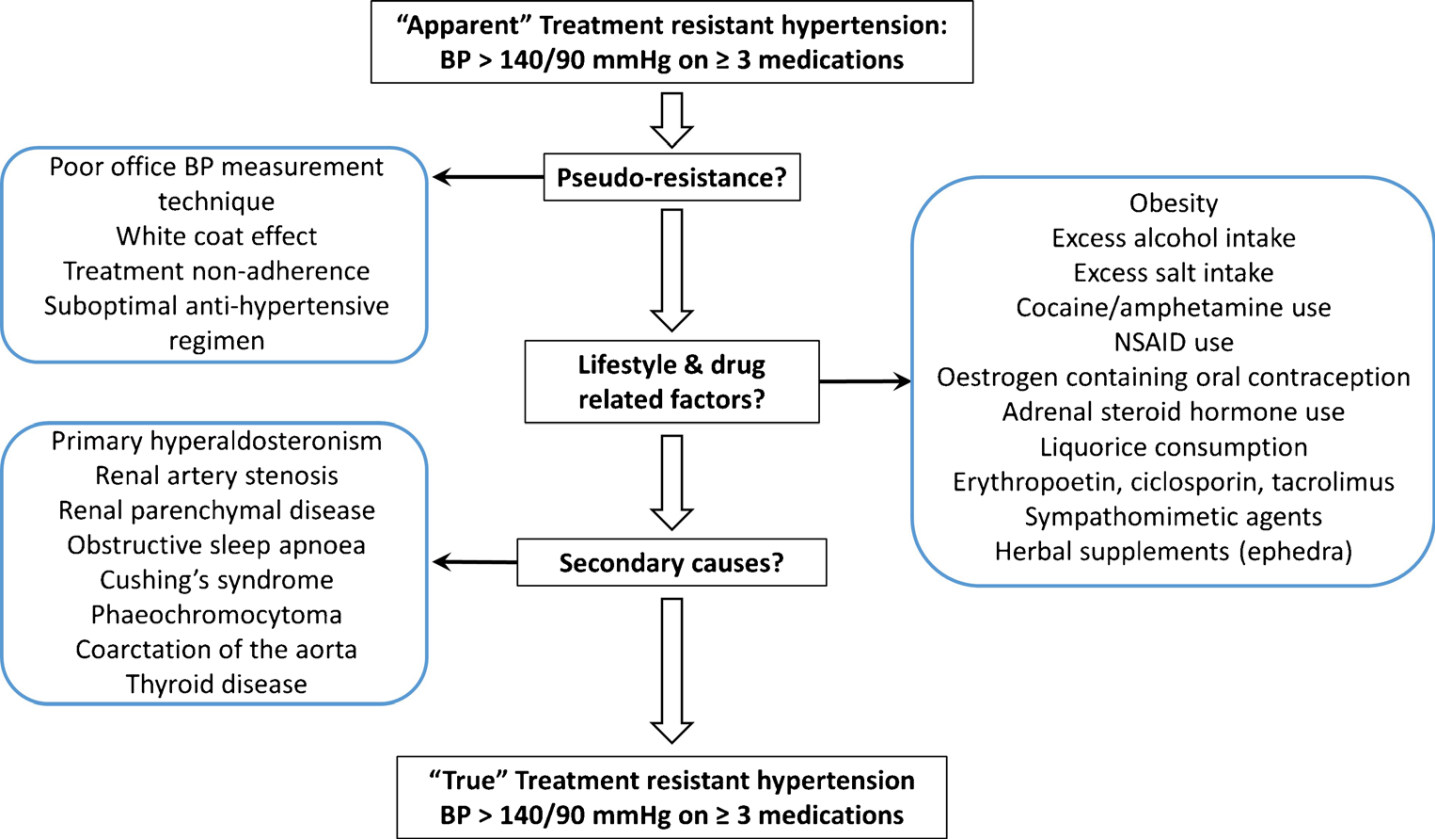
Algorithm for diagnosis of treatment resistant hypertension (TRH). TRH should be considered a provisional diagnosis dependent on adequate remediation of lifestyle and drug related factors and exclusion of secondary causes. Adapted from [4]

Hypertension is the single largest risk factor for death worldwide, accounting for an estimated annual 9.4 million deaths and 7 % of total disability life adjusted years globally in 2010 [1]. Treatment resistant hypertension (TRH), defined as having a blood pressure of ≥140/90 mmHg despite at least 3 antihypertensive drugs, ideally including a diuretic [2], remains a significant problem, estimated to affect up to 8 % of patients identified from registry data using 24-h ambulatory blood pressure monitoring (ABPM) [3]. TRH may be regarded as ‘apparent’ or ‘true’ depending on whether other causes of hypertension have been fully excluded and whether un-remediated lifestyle factors such as obesity and high dietary salt intake have been adequately addressed (Fig. 1).

The optimal drug choice in TRH is not agreed. Observational studies have shown a significant positive association between greater plasma aldosterone levels and blood pressure in both non-hypertensive [5] and hypertensive [6] populations, as well as a greater prevalence of primary hyperaldosteronism in those with TRH [7]. Although multiple contributory causes are likely responsible for TRH, one potential mechanism is the phenomena of aldosterone ‘breakthrough’ whereby aldosterone levels rise to normal levels despite treatment with angiotensin converting enzyme inhibitors (ACEi) or angiotensin receptor blockers (ARB). This occurs in 10 % of patients treated with ACEi/ARBs over 6 months, and >50 % over 1 year, leading to excess sodium retention, hypertension and other adverse cardiovascular effects [8]. This hypothesis has revived interest in the use of mineralocorticoid receptor blockers (MRB), in particular spironolactone and eplerenone, to treat this problem.

The purpose of this article is to critically review the use of MRB in TRH, focusing on evidence published in the last 3 years. It does not consider other approaches to the treatment of TRH, such as renal denervation, or the critical issue of ensuring adherence to treatment.

## Use of MRBs in the Treatment of TRH

Spironolactone, developed in the 1950s, and the epoxy derivative eplerenone, developed in the 1980s, are the two currently available MRBs. Eplerenone has up to 500-fold less affinity for androgen and progesterone receptors compared to spironolactone, reducing the side effects of painful gynaecomastia in men and menstrual disturbances in women. However, eplerenone is a less potent MRB than spironolactone (IC_50 MR_: eplerenone 81nM; spironolactone 2nM) [9], leading to a greater antihypertensive potency of spironolactone than eplerenone [10].

Evidence for the use of spironolactone for the treatment of TRH prior to the last 3 years in observational studies [11, 12] and clinical trials [13–15] is supportive, as is the case for eplerenone [16, 17], although insufficient to alter treatment guidelines. As a result, significant new trials have been published in the last 3 years.

## New Evidence from the Past 3 Years

### Sources and Selection Criteria

A literature search was performed for relevant studies between January 2013 and December 2015 using PubMed, the Cochrane Library and EMBASE with the search terms ‘hypertension’, ‘resistant hypertension’, combined sequentially with ‘spironolactone’, ‘eplerenone’, ‘mineralocorticoid receptor blocker’, and ‘mineralocorticoid receptor antagonist’. Studies were selected according to the criteria of (1) English language (2) human subjects (3) adults (4) meta-analyses, randomized active or placebo-controlled trials, prospective studies, and observational studies with control groups. Using this approach, we identified 7 clinical trials and 2 meta-analyses summarized in Table 1, which will now be briefly discussed. All used spironolactone as the MRB.

**Table 1:** Summary of effects of spironolactone in resistant hypertension in observational and interventional trials between 2013 and 2015

Study	Design	Patients	Treatment	n	Duration (week)	Within group		Between group	
						Δ Baseline BP (mmHg)	*p*	BP difference (mmHg)	*p*
*Abstract only*									
Djoumessi et al. [18]	Single-blind, randomized	3 drugs including diuretic	Spironolactone 25 mg od	9	4	−33^a^	–	−19^a^	<0.001
		Diabetes	vs Alternative	8		−14^a^			
*Published*									
Oxlund et al. [19•]	Double-blind, randomized, placebo-controlled	3 drugs (diuretic not specified)	Spironolactone 25 mg od	57	16	−9.6^b^	–	8.9^b^	<0.001
		Type 2 diabetes	vs Placebo	55		−0.7^b^			
Vaclavik et al. [20]	Double-blind, randomized, placebo-controlled	3 drugs including diuretic	Spironolactone 25 mg od	81	8	−11.5^b^	–	−9.8^b^	<0.001
			vs Placebo	80		−1.7^b^			
Xiaoying Ni et al. [21]	Double-blind, randomized, placebo-controlled	3 drugs including diuretic	Spironolactone 25 mg od	40	12	−11.5^c^	–	−12.5^c^	<0.050
		Dialysis patients	Placebo	36		+0.5^c^			
Rosa et al. [22]	Open-label randomized	3 drugs including diuretic	Intensified drug regimen	54	24	−8.1^c^	<0.001	1^c^	0.360
			Renal denervation	52		−8.6^c^	<0.001		
Verdalles et al. [23]	Observational open-label	3 drugs including a diuretic	Spironolactone 25 mg od	15	24	−24^c^	<0.01	–	–
			Furosemide 40 mg od	15		−13.8^c^			
Williams et al. [24••]	Double-blind, randomized, placebo-controlled crossover	3 drugs including diuretic	Spironolactone	285	12	−14.4^d^		Spironolactone vs Placebo: −10.2^d^	<0.0001
			Doxazosin	282		−9.1^d^		Spironolactone vs Doxazosin: −5.30^d^	<0.0001
			Bisoprolol	285		−8.4^d^		Spironolactone vs Bisoprolol: −5.98^d^	<0.0001
			Placebo	274		−4.2^d^			

^a^Self blood pressure measurement

^b^Mean systolic daytime ABPM

^c^Mean systolic 24 h ABPM

^d^Mean home systolic blood pressure

### Clinical Trials in Abstract Form

Djoumessi et al. [18] compared spironolactone with alternative medication in 17 patients with type 2 diabetes and TRH in a randomized single-blind trial, published only in abstract form thus limiting its interpretation and appraisal. After 1 month, patients on spironolactone showed a significantly greater fall in home systolic BP than those on alternatives, systolic BP falling from baseline in the spironolactone group by 33 mmHg compared with 14 mmHg in the alternative treatment group (*p* < 0.001). No significant changes in plasma creatinine or potassium were observed in either group. Although encouraging in its support for the BP-lowering effect of spironolactone in TRH, this was a small open-label trial without 24-hour ABPM. The alternative treatments were not specified, so no practical conclusion can be drawn.

### Published Clinical Trials

Oxlund et al. [19•] compared the addition of spironolactone 25 mg od or placebo to the treatment of 119 patients with type 2 diabetes and TRH in a double-blind, randomized, placebo-controlled trial. All patients were treated with ACEI and/or ARBs together with diuretics, with 66 % patients in the spironolactone group and 69 % in the placebo group being obese (BMI >30 kg/m^2^).

At 16 weeks, average daytime placebo-corrected BP was reduced by 8.9 (4.7 to 13.2)/3.7 (1.5 to 5.8) mmHg (*p* < 0.001) in the spironolactone-treated group. One patient in the spironolactone group discontinued treatment and three patients required dose reductions due to hyperkalaemia, with one additional patient stopping treatment due to symptomatic hypotension. The strengths of this study are that it used 24-h ABPM in a population with type 2 diabetes, the relatively long duration of 16 weeks and good trial design. The main limitation is comparison with placebo, which precludes comparing how effective spironolactone is against other commonly used antihypertensive medications. In addition, medication adherence was assessed by counting returned tablets rather than biochemical blood or urine analysis. Although the white male predominance may limit generalizability to females and other ethnic groups, obesity and type 2 diabetes are very common comorbidities in our clinic population; thus, overall, this study is supportive for the efficacy and safety of spironolactone in TRH.

The ASPIRANT-EXT trial [25] compared the effect of adding spironolactone 25 mg od or placebo in 150 patients with TRH in a randomized double-blind placebo-controlled trial. At 8 weeks, both systolic and diastolic daytime 24-h blood pressures in the spironolactone group were significantly lower than that in the placebo (−11.5 [±13.4] vs −1.7 [±13.9] mmHg, *p* < 0.001 and −5.6 [±8.5] vs −2.3 mmHg, *p* < 0.001). Serum sodium was significantly lower and potassium significantly higher in the spironolactone group than in the placebo at 8 weeks although no one discontinued treatment due to hyperkalaemia or renal impairment. There was no significant difference in adverse events or serious adverse events between the treatment groups. Strengths of this study include the relatively large sample size and use of 24-h ABPM at baseline and as the endpoint. The major weaknesses include lack of checks on patient adherence prior to enrolment, comparison to a placebo, which does not provide information about comparative efficacy to other antihypertensive agents, and a relatively short treatment duration of 8 weeks. The lack of black and diabetic patients may limit the generalizability of these results. Overall, this study supports the efficacy and safety for the use of spironolactone in patients with TRH albeit not in comparison to other antihypertensive agents.

Xiaoying Ni et al. [21] conducted a prospective randomized, double-blind trial comparing the effects of 12-week treatment with spironolactone 25 mg od versus placebo in 82 patients with TRH on dialysis. When corrected for the change in BP in the placebo group, the average morning BP was reduced by 17.0 (16 to 18)/8 (6.1 to 10) mmHg and the average 24-h BP was reduced by 12.5 (11.2 to 13.8)/7.0 (5.4 to 8.6) mmHg. The strengths of this study include its design and inclusion of diuretics as part of the definition of TRH and longer treatment duration of 12 weeks. The major weakness is, as before, the use of a placebo. Thus while supportive of the beneficial effect of spironolactone in TRH, this study does not tell us what the best treatment is and is not generalizable to the wider population of non-dialysis patients.

Verdalles et al. [23] conducted a prospective observational open-label study comparing the effect of spironolactone 25 mg od or furosemide 40 mg od in 30 patients with TRH attending a nephrology outpatient clinic. Patients with TRH were assigned treatment with either spironolactone or furosemide as per the usual clinical practice of the treating physician. Spironolactone treatment led to a significantly greater reduction in both systolic and diastolic BP at 24 weeks compared to baseline than furosemide (−24 [±9.2] vs −13.8 [±2.8] mmHg for systolic, −11 [±8.1] vs −5.2 [±2.2] mmHg for diastolic, both *p* < 0.01). Furthermore, there was no significant change in eGFR in either group at 24 weeks compared to baseline, with two patients developing mild hyperkalaemia in the spironolactone group though they did not discontinue treatment. The strengths of this study include the use of ABPM at baseline, 12 and 24 weeks to exclude the effect of white coat hypertension, the relatively long duration of 24 weeks, specification of a diuretic as part of the drug regimen as those defined as having TRH, and inclusion of patients with renal impairment, the mean eGFR being 55.8 [±16.5] mL/min/1.73 m^2^. Although this study is supportive of the use of spironolactone in patients with renal impairment and TRH, it has serious limitations that restrict the wider conclusions that can be drawn. Most importantly, this was a small open-label non-randomized observational study which inherently cannot exclude selection bias between the two treatment groups. The method by which patient adherence was checked prior to enrolment was not specified, and the use of furosemide as the sole comparator to spironolactone did not allow the effects of alternative treatments, such as β blockers, to be compared. Thus, overall, this study adds little to support the use of spironolactone in TRH.

While the previously discussed trials have compared spironolactone to either placebo or alternative medical treatments, Rosa et al. [22] compared spironolactone to renal denervation therapy in 106 patients with TRH in a randomized open-label study. At 24 weeks there was a significant fall in mean systolic BP measured by ABPM in both treatment arms (−8.6 mmHg with renal denervation, *p* < 0.001 versus −8.1 mmHg with intensified pharmacotherapy, *p* = 0.001), but no significant difference between the two groups (*p* = 0.36). At 24 weeks, compared to baseline, the proportion of patients treated with an MRB in the intensified pharmacotherapy group increased from 25 to 61 % (*p* < 0.001), while there was no significant change in the number of patients prescribed amiloride, β blockers, α blockers and centrally acting agents. The between-group 24-h systolic BP difference, however, remained nonsignificant (*p* = 0.46) when the number of drugs and MRB use were adjusted for. Disregarding the renal denervation arm of this trial, controversial following failure of the SIMPLICITY-3 trial to show a benefit of renal denervation [26], the use of ABPM, the long treatment duration and use of drug assays to confirm adherence are the strengths of this study. Unfortunately, although spironolactone does appear to have been the most commonly added drug in the intensified pharmacotherapy arm, which did show a significant reduction in BP from baseline, its use was not the main focus of this study which somewhat limits further interpretation of these results in the context of specifically addressing the efficacy of spironolactone in TRH.

The most important and recent new evidence for the effectiveness of MRB in TRH is the PATHWAY-2 study by Williams et al. [24••] published in the *Lancet*. In this randomized double-blind crossover trial, patients with hypertension resistant to 3 drugs including a diuretic were sequentially treated with spironolactone (*n* = 285), an α_1_ blocker doxazosin (*n* = 282), β blocker bisoprolol (*n* = 285) and a placebo (*n* = 274), each for 12 weeks, in random order, with 230 patients completing all treatment arms. Spironolactone treatment led to significantly greater mean reductions in systolic blood pressure compared to placebo (−10 [−11.7 to −8.74] mmHg, *p* < 0.001), doxazosin (−5.64 [−69.1 to −4.36], *p* < 0.001) and bisoprolol (−5.98 [−7.45 to −4.51] mmHg, p < 0.0001) on home systolic BP readings taken at the final visit of each treatment cycle. The magnitude of BP lowering with spironolactone was inversely proportional to plasma renin concentration, an association not observed with either bisoprolol or doxazosin, supporting the role of sodium retention, volume expansion and low plasma renin in TRH. As well as clearly demonstrating the superiority of spironolactone as the most effective add-on medication in TRH compared to bisoprolol and doxazosin, spironolactone was shown to be safe and well tolerated with no greater discontinuation rate due to renal impairment and hyperkalaemia with spironolactone than the other treatments.

This trial has a number of important design strengths that radically improve upon all previous published studies. Most importantly, it is the first trial to compare the effect of spironolactone to other commonly used antihypertensive drugs as well as placebo. In addition, home BP monitoring was used as part of screening for eligibility to assess adherence by measurement of BP 6 h after directly observed therapy, as well for the primary endpoint of average of home systolic BP recorded throughout the treatment cycle. This likely reduced the potential confounding influence of non-adherence which has previously been found to be an important factor in TRH [27•] as well as allowing the exclusion of white coat hypertension. Other strengths included a standardized definition of TRH to include a diuretic in all enrolled patients, a longer 12-week duration of treatment, measurement of serum ACE to assess if patients were adherent with ACE inhibitor medication, and a crossover design, which enabled comparison of the effectiveness of medications in individual patients.

Limitations of the study include exclusion of patients with moderate renal impairment (eGFR < 45 mL/min/1.73 m^2^), lack of a washout period between switching treatment group, exclusion of type 1 diabetics and a low proportion of black patients. Overall, however, these results provide robust new evidence supporting the use of spironolactone in TRH, for the first time giving clinicians clear guidance on the best treatment among commonly used alternatives.

### Meta-Analyses

Two meta-analyses combining the results of several studies of spironolactone in resistant hypertension conducted over the previous several years were published in 2015. Neither included the PATHWAY-2 study.

The meta-analysis by Dahal et al. 2015 [28] included 15 studies in total published between 2002 and 2013 comprising 3 randomized controlled trials (135 patients in total treated with MRB vs 136 control), 1 non-randomized comparative study and 11 observational studies (898 patients in total treated with MRB). Only 1 study exclusively used eplerenone, 12 used spironolactone, and 2 studies used both eplerenone and spironolactone. Follow-up varied from 5 to 40 weeks. Meta-analysis of the combined results of the 4 fcomparative studies demonstrated that spironolactone reduced pooled mean office systolic BP by 24.3 [8.65 to 39.9] mmHg (*p* = 0.002, *I*^2^ = 95 %), while meta-analysis of the observational studies showed a pooled mean reduction in office systolic BP of 22.7 mmHg (18.2 to 27.3, *p* < 0.00001, *I*^2^ = 96 %). MRB were not associated with an increased risk of hyperkalaemia compared to placebo in the comparative studies (risk ratio = 2.93 [0.5 to 18.1], *p* = 0.25, *I*^2^ = 0 %) although an increased risk was noted in the observational studies.

Hongyin Guo et al. [29] systematically reviewed 8 studies published between 2002 and 2013, comprising 5 observational (1806 patients in total, all treated with spironolactone) and 3 (270 patients in total, 135 treated with spironolactone) randomized placebo-controlled trials encompassing 2051 patients in total involving patients with TRH. Follow-up duration was between 4 and 24 weeks. Analysis of the combined results of the 3 controlled studies showed spironolactone to reduce pooled mean systolic BP by 20.6 (36.5 to 4.7) mmHg compared to placebo (p < 0.00001), while analysis of the 4 observational studies which had 24 weeks of follow-up showed a fall in pooled mean systolic BP of 20.7 (25.6 to 15.8) mmHg, *p* < 0.00001, after spironolactone treatment.

High levels of heterogeneity were noted between both controlled and observational studies (*I*^2^ = 98 % and *I*^2^ = 81 %, respectively), meaning these results just as with Dahal et al. 2015 [28] should be interpreted with considerable caution. Although the consistent direction of benefit of spironolactone in TRH is reassuring, they both highlight the poor quality of the evidence base comprised of small placebo-controlled trials and observational studies prior to the publication of PATHWAY-2. In addition, they did not address the important clinical question of identifying the most effective drug for TRH.

## Discussion

Until the publication of PATHWAY-2, the overall quality of evidence in support of the use of MRB in TRH has been poor despite a general concordance over its positive therapeutic effect in both observational and interventional studies. Methodological issues of inadequate sample sizes, short trial durations and use of a placebo as a comparator have all beset previous work. As a consequence, support for the efficacy and safety of MRB in TRH has been weak, reflected in the low grade of its recommendation in current major treatment guidelines.

PATHWAY-2 has been vital in finally addressing the limitations of previous studies in particular by using active comparators of widely used antihypertensive medications in addition to a placebo. Furthermore, it provides the first biological justification for use of MRB in a non-primary hyperaldosteronism population by the analysis of the BP response to different treatments with respect to plasma renin levels, indicating that sodium retention may play an important role in TRH. PATHWAY-2 is an excellent example of the importance of specialist society-led and charitably funded drug trials not driven by a commercial imperative.

However, further questions remain to be resolved. In particular, we lack sufficient data on the use of MRB in patients with mild to moderate renal impairment, and in diabetic and black patients. In addition, we do not yet know whether treatment with higher doses of thiazide and thiazide-like diuretics, or potassium sparing diuretics such as amiloride, may be equally effective.

As always, translating the results of clinical trials into day-to-day care often proves less than straightforward, and, despite routinely using spironolactone in our specialist hypertension service for several years, significant challenges remain. Managing patients with multiple drug intolerances, with renal impairment and significant lifestyle issues all remain common problems we see each week and which present important barriers to good hypertension control. Indeed, a recent study using routine screening for medication adherence confirmed high levels of medication non-adherence in patients attending a specialist hypertension clinic [27•]. This only serves to highlight that while drug treatments such as spironolactone can be effective, they are only part of a holistic approach encompassing education and motivation to achieve lifestyle change and good drug adherence.

## Conclusion

We believe sufficient evidence has now accumulated to recommend spironolactone as the first choice drug for TRH and hope clinical guidelines and algorithms will soon be updated to reflect this important new evidence.
